# Physical Activity Discussions as the First Step Towards Movement in Non-Curative Cancer Care: A Qualitative Study

**DOI:** 10.3390/healthcare13172126

**Published:** 2025-08-26

**Authors:** Jodi Langley, Grace Warner, Christine Cassidy, Robin Urquhart, Melanie R. Keats

**Affiliations:** 1 Faculty of Health, Dalhousie University, Halifax, NS B3H 4R2, Canada; grace.warner@dal.ca (G.W.); ccassidy@dal.ca (C.C.); melanie.keats@dal.ca (M.R.K.); 2 Beatrice Hunter Cancer Research Institute, Halifax, NS B3H 0A2, Canada; robin.urquhart@dal.ca; 3 Department of Community Health and Epidemiology, Dalhousie University, Halifax, NS B3H 4R2, Canada; 4 Department of Medical Oncology, Nova Scotia Health, Halifax, NS B3S 0H6, Canada

**Keywords:** physical activity, non-curative cancer, qualitative, theoretical domains framework

## Abstract

Background: Due to advances in technology, non-curative cancer patients are living longer with the burden of the disease. Physical activity (PA) has value as a supportive service for individuals at any point on the cancer care continuum, including advanced cancer. Objectives: The objective of this study was to understand how oncology care providers (OCPs), family/friend caregivers, and health system decision makers can include conversations of PA into care for those living with non-curative cancer. Methods: We conducted 24 semi-structured interviews guided by the Capability, Opportunity, Motivation–Behaviour (COM-B) model and further refined them using the Theoretical Domains Framework (TDF). The data were then analyzed using a directed content analysis, with a codebook generated based on the TDF. Results: Findings highlighted the need for targeted education and effective communication to address knowledge gaps, motivation-enhancing strategies to overcome emotional barriers, and streamlined referral processes to address systemic barriers to PA across the cancer care journey. Conclusions: There is an ongoing need to support individuals in living well, which involves not only addressing their immediate cancer diagnoses but also promoting overall health. This can be performed through PA, supported by their OCPs. This study further emphasizes the inclusion of PA discussions in cancer care.

## 1. Introduction

Cancer is one of the leading causes of death in Canada, with two in five Canadians diagnosed with cancer in their lifetime and one in four dying from the disease [1]. Due to advances in treatment and technology, individuals are living longer with the burden of disease. However, some may never move into survivorship care. The medical management of people living with non-curative cancer can be all-consuming, and individuals often feel distressed, which impairs their quality of life (QOL) [2]. One way that patients can potentially counteract these feelings of distress and optimize QOL is to maintain and/or increase their physical activity (PA) levels [3]. However, PA is still underutilized as a supportive service. Many patients do not meet the guidelines for PA and therefore may not be reaping the health benefits associated with regular, sustained PA behaviour [4].

Non-curative cancer patients warrant specific attention, given the nature and progression of the disease. There is evidence to suggest that PA offers more than just health benefits; it also has therapeutic benefits [5]. However, there is still low awareness among oncology care providers (OCPs) about these benefits, and OCPs are not utilizing the teachable moments that come with a cancer diagnosis to increase patient receptivity to becoming more active [6]. There are unique issues for individuals living with non-curative cancer, with a focus on care focusing on QOL, physical functioning, and symptom management; they also present with a different set of psychosocial challenges [7]. These and other challenges may prohibit non-curative cancer patients from seeking out, accessing, and participating in healthy behaviours, including formal PA programs and initiatives. A key barrier is that OCPs themselves may not be aware of or confident in discussing PA with those living with non-curative cancer, which limits opportunities for meaningful referrals or encouragement. Individuals living with non-curative cancer may also face logistical, emotional, and cultural barriers, including but not limited to transportation issues, fatigue, stigma, or a lack of culturally safe or personally relevant programming [8]. Additionally, some individuals may prefer to be physically active alone or with loved ones rather than joining structured programs. This is especially true if they feel that formal environments do not align with their values, energy levels, or desired level of social interaction. Given the clinical benefits of being physically active for those living with non-curative cancer, we must understand how OCPs can have clinically meaningful conversations around PA and support patients where they are on their PA journey.

There is an awareness that the patient–provider relationship is impactful, so it may be logical to start the discussion around PA with the OCP. However, there are three key reasons why clinicians may not discuss PA with patients: (1) OCPs are unaware of PA recommendations for people living with and beyond cancer [9,10], (2) there is a lack of clear ownership over who should discuss PA and its benefits with people living with and beyond cancer, and (3) PA is not embedded in standard care, which results in limited continuity across clinical sites. Some evidence suggests that healthcare providers who are aware of lifestyle guidelines are more likely to offer advice on lifestyle factors [11,12]; however, other studies highlight that knowledge alone is insufficient [13,14]. Effective behaviour change communication requires more comprehensive training to support meaningful, patient-centred conversations [14].

In cancer care, there is a growing need to support OCPs in conducting effective and focused clinical visits with patients. When exploring a new way for clinicians to discuss PA as part of the standard of care, researchers recommend using a theory-driven approach to identify the barriers and facilitators influencing the desired behaviour. One such approach is the Behaviour Change Wheel (BCW), which incorporates frameworks such as the Capability, Opportunity, Motivation–Behaviour (COM-B) model and the Theoretical Domains Framework (TDF) to guide the design and delivery of sustainable and effective interventions [15,16]. These tools are particularly relevant to identifying how to promote meaningful conversations between OCPs and patients about PA. In clearly defining the components of the target behaviour, a theory-based approach also helps determine which strategies or tools may be most effective. The COM-B model and TDF have already been applied in oncology settings to explore how PA guidelines can be better implemented in practice, offering valuable insights into bridging the gap between evidence and care delivery [10].

The primary purpose of this study was to gain a deeper understanding of the barriers and facilitators that OCPs, patients, family/friend caregivers, and health decision makers encounter when discussing PA with people living with non-curative cancer during clinic visits, including but not limited to personal and systemic factors that are at play during appointments. By examining the perspectives and experiences of these key individuals, this study seeks to identify the challenges that may hinder such conversations, as well as the factors that support and encourage them. Additionally, this study aimed to uncover practical and effective strategies that OCPs can use to introduce and promote PA in a clinical setting, to enhance patient care and encourage long-term health outcomes.

## 2. Methods

### 2.1. Study Design

This study is part of a larger project aimed at creating a theory-informed intervention to enhance PA discussions in clinical care for individuals living with non-curative cancer in Nova Scotia, Canada [17]. A qualitative descriptive design was used [18,19] to conduct semi-structured interviews with patients, OCPs, family/friend caregivers and health decision makers. The study was approved by Nova Scotia Health Research Ethics Board (REB#1029051).

### 2.2. Theoretical Approach

The BCW integrates 19 existing behaviour change frameworks and provides a systematic, theory-driven framework for designing interventions, grounded in the COM-B model [15,20]. It guides users through analyzing the nature of a behaviour, identifying the mechanisms that influence it, and selecting appropriate interventions and policies to target those mechanisms effectively. An extension of the COM-B model, the TDF, further refines behaviour analysis by categorizing influencing factors into barriers and facilitators [16,21]. It further enables the identification of specific psychological and contextual determinants, such as social influences, emotional responses, reinforcement, behavioural regulation, and physical skills that impact behaviour. In this study, the TDF was applied in conjunction with the BCW/COM-B to explore barriers and facilitators influencing discussions about PA among individuals with non-curative cancer in clinical settings. Additionally, determining what system-level supports might assist care teams and health decision makers in better integrating PA conversations into clinical visits.

### 2.3. Participants/Recruitment

The study team recruited participants using various methods from all areas of Nova Scotia. Patients and family/friend caregivers were provided with the research team’s contact information by a member of their care team at the Nova Scotia Cancer Center and could reach out directly to the research team if they wanted to participate. Several patients were previous participants in a clinical trial conducted at one of the senior author’s (MK) exercise oncology labs. The study team recruited OCPs through their work email, both individually and in groups, as well as through word of mouth. Potential participants were given the lead author’s email to contact when it was convenient. Purposive and snowball sampling [22] was used to target multiple professional roles within Nova Scotia Health, involving OCPs and health decision makers, to investigate a broad range of perspectives and patterns concerning PA within oncological care and the healthcare system. These targeted individuals included oncologists, oncology nurses, social workers, surgeons, and other allied health professionals. Health decision makers included leaders in the cancer care program, middle managers, program directors, and key individuals responsible for directing healthcare initiatives in Nova Scotia.

### 2.4. Inclusion and Exclusion Criteria

Participants were informed of the methods and study design verbally and in writing before providing verbal informed consent. The primary author screened participants to determine eligibility. Participants had to meet one of the following criterion: patients diagnosed with a non-curative diagnosis and have a predicted life expectancy of >3 months; family/friend caregivers currently caring for someone who has been diagnosed with a non-curative cancer; OCPs and health decision makers currently or previously had cared for patients with non-curative cancer or oversee programs for people living with non-curative cancer and work for Nova Scotia Health or Department of Health and Wellness or have a role in providing formal support. All participants had to reside in Nova Scotia, be over 18 years of age and be able to speak and read in the English language (to provide informed consent).

### 2.5. Data Collection

A semi-structured interview guide based on the BCW-COM-B and TDF frameworks was created [16] and built upon previous work with glioblastoma patients [17]. The interview guide was used to explore participants’ beliefs about PA discussions in clinical practice and focused on the barriers and facilitators to having these discussions. Interviews probed participant narratives to understand the factors that affected OCP and patients’ and family/friend caregivers’ abilities to engage in PA conversations during clinical visits. Questions covered perceptions of PA, organizational and health system determinants, barriers and facilitators to PA conversations and details of what occurs during appointments. Open discussion was encouraged to identify and explore evolving themes. Semi-structured interviews provided flexibility to examine specific issues in greater depth as needed. OCPs and health decision makers were interviewed first, followed by patients and family/friend caregivers. JL conducted all interviews over Zoom, the phone, or in person between January 2024 and May 2025. Interviews ranged in duration, lasting between 17 and 94 min, with an average length of 44 min. An external transcriptionist transcribed all audio-recorded interviews verbatim. Recruitment continued until all targeted groups were represented and no new themes of analytical relevance emerged within the constraints of the project timeline. After the team completed preliminary interviews and analyzed the coded data, four additional repeat interviews were conducted with each participant group.

### 2.6. Data Analysis

Semi-structured interview transcripts were combined to create one complete dataset and then were read and re-read by the lead author (JL) to develop a working understanding of what was happening during clinical visits, how individuals felt about PA discussions, barriers/facilitators to discussions around PA, as well as PA behaviour and other disease-specific factors. JL is a PhD candidate at Dalhousie University; she took time to reflect on her role within this project and the greater idea of exercise oncology. She is innately aware of her role in the analysis process and working through her own bias of PA as being part of the standard of healthcare. In discussing data and themes, she spent time working with people who are not in the exercise oncology field, including GW, CC, and RU, to minimize bias. Data were then analyzed using direct content analysis [23] followed by inductive thematic analysis [24]. We used a deductive approach to generate the codebook using the 14 TDF domains and using previous TDF literature [16,25,26] to define each domain concerning the behaviour at hand. The research team reviewed and refined the initial codebook to ensure clarity, consistency, and alignment with the study objectives. To guide the analysis, the TDF acted to identify specific psychological, social, and contextual factors influencing discussions about PA within clinical care with non-curative cancer patients. The TDF provided a structured lens for coding and organizing the data at a granular level, allowing for a detailed overview of barriers and facilitators across individual, social, and environmental domains. To examine how these domains interacted, they were double- and triple-coded to identify patterns of co-occurrence and influence across the coded data, enabling the exploration of how intrapersonal, interpersonal, and environmental/systemic conditions collectively shaped engagement in PA discussions.

A single researcher (JL) coded all data using NVIVO 14 [27], and validity was supported by peer-reviewing themes with participants (follow-up interviews) and co-working with a patient partner and senior scientists (GW, MK) [28]. Data were synthesized into broader, overarching themes aligned with the COM-B model. The COM-B framework served as a higher-order categorization tool, enabling us to distill the detailed TDF constructs into core behavioural components that underpin PA counselling in clinical care. This process was iterative, ensuring that each theme aligned with the COM-B model, was derived from the data, and was linked to relevant TDF domains. This maintained the use of both frameworks, utilizing the TDF to understand conceptual specificity and the COM-B model to emphasize behavioural mechanisms. This allowed the thematic structure to reflect both the nuance of individual experience (via TDF) and the broader behavioural drivers (via COM-B), supporting a theory-informed understanding of intervention targets [15,16]. This approach enabled the categorization of qualitative data within a behaviour change framework while providing greater specificity through the TDF domains. The primary author (JL) generated themes around key concepts from the interviews and these themes were continually refined, and new themes were added as new data were presented. Following this, all authors reviewed the themes, during which clarification of the themes was discussed. In line with an iterative approach, follow-up interviews were conducted with four individuals to refine emerging themes and gain a deeper understanding of the data, leading to a more nuanced interpretation [29].

## 3. Results

Twenty-four individual interviews were conducted with non-curative cancer patients (n = 5), family/friend caregivers (n = 2), OCPs (n = 11), and health decision makers (n = 6), along with four additional follow-up interviews (one individual from each subgroup). OCP interviews were conducted with five oncologists, two oncology nurses, one surgeon, and three allied health professionals (e.g., cancer care navigator and social worker). Health decision makers were in a unique position, with all but one having previous clinical or “floor” (direct patient care) experience before transitioning to a managerial or leadership position. Themes are described below, including how OCPs, patients, family/friend caregivers, and health decision makers conceptualize and act when it comes to PA discussions in cancer care. Themes were first laid out following the TDF; however, due to complexities in overlapping determinants, larger COM-B themes were created to highlight the behavioural drivers, allowing for a greater understanding.

### 3.1. TDF Factor Influencing PA Discussion

Table 1 highlights each TDF domain, its definition, a specific theme, and a representative quote. The TDF domains were defined using the original definition by Michie et al., 2011 [15]. Each TDF domain is defined and then paired with inductive themes derived from the interviews. As not all domains were represented in the data, the analysis defines and highlights only 11 of the TDF domains that were identified.

### 3.2. Overarching Themes Aligning with the COM-B Model

There is a complex interplay of knowledge, skills, beliefs, emotions, and environmental factors that shape how OCPs, patients, family/friend caregivers, and health decision makers perceive and engage in discussions around PA that came out of the TDF analysis. This led to the development of overarching COM-B themes. Understanding the factors that influence the integration of PA discussions and referrals into cancer care is essential for improving patient outcomes. The analysis revealed three overarching themes aligned with the COM-B components of psychological capability, reflective motivation, and physical opportunity. These were systematically explored by examining the cognitive, social, and environmental determinants that impact OCPs’ behaviours.

In examining TDF themes across the domains, especially knowledge, skills, memory/attention, and beliefs about capabilities, a broader barrier related to psychological capability was identified. OCPs described limited knowledge about how to integrate PA into cancer care, uncertainty about what advice to give, and difficulty recalling specific PA guidelines during clinical interactions. Participants highlighted these challenges by emphasizing the need for more targeted and embedded education strategies. Concerning reflective motivation, the analysis that PA discussions were linked to emotions, beliefs about consequences, and intention highlighted that both OCPs and patients experience emotional barriers, such as fear of harm, guilt, or uncertainty. Despite these challenges, participants expressed a clear motivation to overcome these emotional barriers. They were committed to addressing these challenges through improved communication, relationship-building, and system-level strategies that foster more meaningful and personalized PA engagement. Themes from the domains of environmental context, social influences, and reinforcement led to barriers related to physical opportunity. These included inconsistent referral pathways, a lack of prompts within electronic medical records (EMRs), and systemic fragmentation that delayed or prevented patients from accessing PA support. Participants expressed a strong desire for more streamlined and integrated systems. Table 2 presents all three themes following the COM-B model. These behavioural drivers via COM-B offer a nuanced understanding of the challenges and opportunities in promoting PA as a component of cancer treatment for those living with non-curative cancer.

### 3.3. Psychological Capability

Effective communication and targeted education are essential to addressing knowledge gaps about PA. These targeted education programs can help influence beliefs, supporting informed decision making, thoughtful engagement, and behaviour change. They can also be affected by (1) health decision makers influencing OCPs and (2) OCPs influencing patients.

This was highlighted by a surgeon who discussed the need for patients to be educated on how they must be strong to withstand surgery.

*“But I say, and you have to be strong for the treatments. So before the treatment start, you’ve got to do this so that you’re strong because you’re going to lose some of your muscle mass. But if you can build that up beforehand then you’ll tolerate it better, and have a better quality of life during that time.”*

OCP 03

OCPs are also aware that the term “exercise” can be overwhelming for patients, and they try to use fewer intimidating terms to relate to them. They have an understanding that even changes in terminology are an important process when talking with patients about exercise and/or physical activity and/or movement.

*“This actually just came up. I’ve been doing this a long time, and this just came up within the last year… And so I remember just this year like asking… I would always ask women when I’m doing their follow-up calls post-op, Have you been able to do your exercises? Because it’s important, right, to keep mobility. And I’ve had a few… I got the strong vibes from them that it was exercise, and they had an aversion to exercise. And I was like, Ha. Like it hadn’t even occurred to me like just calling it exercise… was a negative for them, and that they weren’t necessarily going to be doing their exercises. But when we talk through, Well, how are you moving, yes, they’re putting their dishes in the cupboard, and they’re reaching, and moving, and doing. But yeah, that was an aha for me. And like I said, I’ve been doing this quite a long time. So I was like, okay, yeah, the connotation of exercise really was off-putting for a couple of women.”*

OCP 07

OCPs discussed meeting patients where they are, and not thinking of PA as a big undertaking, but rather as small, digestible steps along the way.

*“And what I always say to my patient, especially my sick ones, is don’t go and do a kilometre. Like I say, How far from here to your mailbox, if they live rurally? And I say, “You don’t have to do it three times all at once. Do it once, and then do the same thing frequently, and build up that way,” is what I usually tell them. But yeah, something… Yeah, how do you reach more people? And even if you can reach 1 in 4, right?”*

OCP 04

Further, many health decision makers discussed not only the knowledge gap in patients, but also in providers.

*“I still think that there’s an education gap there. And I don’t see that everyone truly understands the actual survival benefit. Because I mean that’s ultimately what guides us in many cases for our treatments.” *

Health Decision Maker 06

Health decision makers noted their role in pushing initiatives forward with nurses and other staff, with the understanding that staff care about how these initiatives will affect patients and have a desire to hear from them.

*“They don’t care about me saying, Here’s this program. They want to know how it’s going to impact the patients.”*

Heath decision maker 19

### 3.4. Reflective Motivation

There is an understanding that emotional barriers impact engagement with PA. OCPs and health decision makers are motivated to address these challenges by prioritizing process improvements and support strategies that foster motivation and make PA more accessible and meaningful for patients.

Patients often reflected on their journey in cancer care, and the notable steps that led them to where they are now, with an understanding that encouraging others to do the same may be beneficial.

*“But I’m still here today. I fought it with everything they gave me to fight it with. And the steroids that they put me on was ruining my thinking and stuff. And now it’s coming back. It’s a lot better than it was… And it’s more exercise. And I find that whatever I do with this exercise, it slows the cancer right down. And I try and tell people that.”*

Patient 11

Additionally, there is an understanding among OCPs of who the patient is and how they can work with them to determine what activity may be best suited for them.

*“So it’s really trying to get a sense of who they are. And like a lot of my patients who live more rural, they might be quite active. Like they’re out gardening and doing those types of things. Whereas they may not be as interested in organized programs. So it’s good to know your patients.”*

OCP 23

OCPs are doing their best to work with patients and to help them navigate not only the cancer itself, but the system around it. OCPs explained that they wanted to help patients not only see the problems happening but also help them avoid other potential issues.

*“…you have a patient who says it’s like they’re climbing a mountain, and someone’s throwing boulders at them now, or are dropping trees in front of them. And so I said what I would love is that I get to the tree first and divert it, and that they never see that boulder even rolling down the hill.”*

OCP 07

Health decision makers recognize the importance of how programs should be communicated and presented, with a particular emphasis on highlighting patient narratives, as this will influence the beliefs, engagement, and motivation of both them and frontline staff.

*“And I was like people can present about their programs all they want, and I’m like it sounds like a good program. But when you hear, you know, the like first person of like what it did for them, I was like, wow, this is… It was obviously impactful”*

Health decision maker 18

### 3.5. Physical Opportunity

There are identified environmental and systemic barriers to engaging patients with PA support, and an emphasized need for streamlined or integrated referral processes to enhance accessibility and allow patients to start PA at any point of their cancer care journey.

When discussing what a successful provider education program would look like for OCPs, OCPs expressed that they were discouraged by all past programs and the limited success of any supportive service or program implementation with the Nova Scotia Cancer Care Program.

*“I just don’t think… I don’t even know if there’s that many, to tell you the truth, [interviewee]. Like I wouldn’t even be able to tell you what’s… Like do you think anything’s succeeded?”*

OCP 13

Health decision makers understand that OCPs operate in a complex environment with many constraints, and sometimes, reminders may be a helpful tool to prompt reactivation or presentation of programs.

*“… we meet once a month as a business meeting and say, “Oh, the Sunshine Room [Resource room for patients] is back open. Here’s what they offer.” You know, just to get little tidbits out there… I think that’s the hardest piece, is like the knowledge dissemination”*

Health decision maker 05

Health decision makers are aware of the reality of the clinical space, and that the role of an OCP may be to raise awareness about PA, but then refer to the proper individuals who can have a more effective and comprehensive discussion, while eliminating barriers such as cost.

*“… where the nurses don’t need to be counselling patients on exercise, they just need to be saying, you know, exercise and some physical activity could really help. With some canned language, you know, like can help with this, this and this. And have it embedded in their initial conversations with patients. And, would you like me to send a referral on to our exercise physiologist who can help explain what’s involved, and how you might do that, and what the options are? And it’s free.”*

Health decision maker 21

## 4. Discussion

In this study, using the BCW and incorporating the COM-B model and the TDF, we aimed to understand the barriers and facilitators that OCPs and health decision makers face when discussing PA during clinic visits. In exploring the perspectives of patients and family/friend caregivers, this study endeavoured to identify what hinders or supports these conversations and to uncover practical strategies OCPs can use to promote PA in clinical care to enhance patient outcomes. Across all participants, there was a shared desire to make care more patient-centred and evidence-based, and to create space for meaningful discussion about PA during clinical encounters. However, systemic barriers continue to impede this goal. These findings highlight three key areas for action: (1) communication pathways are vital for change, (2) emotional support is crucial, and (3) systems need to facilitate access to PA supports. Targeted education and communication strategies are needed to address persistent knowledge gaps and support informed decision making at multiple levels of the cancer care system. Emotional barriers remain significant, underscoring the importance of motivational approaches and patient-centred care strategies that make PA feel meaningful and manageable. Finally, institutional change is necessary to streamline referral processes and embed PA into standard care models; without this structural support, even the most committed OCPs struggle to prioritize PA promotion, further widening the gap between evidence and practice.

There was a distinct similarity and difference regarding the communication pathway and how PA was discussed when a health decision maker discussed the benefits of an OCP versus when an OCP discussed PA with a patient. Effective and educational communication is key, with health decision makers making a point of aligning this education with OCPs’ professional and personal values. The focus on patient wellbeing, time efficiency, and evidence-based practice can be achieved through shared goal setting, values-based education and using patient testimonials [30]. Behaviour change with healthcare providers is known for being complex [31], and proposed ways to change their behaviour are the use of clinic champions and team-based care models. It has been impactful to empower nurses to lead conversations about physical inactivity [32]. A key foundation of the aforementioned intervention was the supportive and motivating conversations patients had with nurses, which centred around patient needs and fostered relationship building. Nurses were encouraged to offer meaningful challenges and practical ideas to work with individuals on their PA journey. Workshops and training can help increase the ease of these conversations, which in turn can boost confidence and ability [33]. It is essential to consider how to create resources (e.g., workshops, pamphlets, and question prompt lists) around the benefits of PA, with communication focused on tolerating treatment better and enhancing/improving QOL. Inversely, when health decision makers communicate with OCPs, the communication tends to focus more on raw data, followed by survival benefits, and then real-world stories about the positive effects. Although these interpersonal characteristics are critical when it comes to communication, it cannot be overstated how they are paired with healthcare system-level support.

The healthcare system is incredibly complex [34,35], and patients and family/friend caregivers are expected to navigate the cancer care system while dealing with the emotional distress of non-curative disease. OCPs have a role in not only explaining the medical complexities of disease but also moving and preventing obstacles to accessing care, so patients do not have to navigate that on top of their emotional distress. PA can help with distress, by reducing anxiety, depression, cancer-related fatigue and sleep disturbances [36,37]. In addition, addressing distress can offer a sense of agency during an uncontrollable diagnosis and help with reframing the physical body to be capable and resilient [38]. Incorporating PA into existing referral systems may help increase the likelihood of patients becoming physically active throughout their diagnosis, with OCP support from the initial diagnosis through treatment.

In cancer care, different OCPs take on various roles, with medical oncologists typically being the team leader throughout the process. Due to this leadership role, they may need to have the highest motivation to discuss PA, but may not always be the ones to discuss it with patients. Reflective motivations in the BCW entail the conscious processes that influence behaviour, including planning, decision making, and evaluating the consequences of actions [15]. In areas where there is a culture of PA promotion and awareness from health decision makers and other team leaders, and actionable ways (e.g., referral forms and discussion guides) to complete this, there is an increase in PA promotion from providers to patients, leading to marked improvements with PA promotion [39]. As seen with the glioblastoma population, the uptake of PA discussions was rooted in other OCPs knowing that the oncologist was leading a research trial on PA, which then offered a direct and trusted referral source, and reassurance that PA was safe for the population [17]. Further, having someone on the care team with PA knowledge who discusses PA directly with patients during clinical visits has the potential to increase the likelihood of patient uptake by enabling a conversation with a content expert. The team may have more casual conversations and follow-up questions with patients because they know someone is the content expert on this topic. Having a culture of PA in care can be an effective way to increase PA discussion, but it may also have a trickle-down effect by giving OCPs the confidence to be more active themselves [40]. When healthcare providers have a personal experience with PA, they are more likely to discuss PA, and when healthcare providers are more physically active, they have reduced psychological stress and increased sleep quality [41]. Further, physicians who meet PA guidelines more frequently counsel patients on exercise [42], are likely to see themselves as role models for their patients, and tend to promote PA more in clinical care [43]. Further, staff who are more active themselves may be better informed about PA resources and therefore more likely to discuss and/or refer to PA resources [9]. These intrapersonal and interpersonal barriers are important considerations when empowering OCPs to discuss PA in clinical care, but there also needs to be robust system-level supports.

The healthcare system faces environmental and systemic challenges, including siloed services, administrative burdens, and fragmented care pathways. OCPs often navigate high workloads, emotionally distressing environments, and limited time for patient counselling. This makes the implementation of supportive care services, such as PA promotion, difficult. While OCPs in this study felt comfortable discussing PA informally, they lacked knowledge of PA guidelines and actionable ways to counsel patients, despite evidence that patients want to hear about PA from their oncologist [44]. Patients value receiving PA guidance, highlighting a clear gap between patient expectations and clinician preparedness. There is a lack of pragmatic tools to help clinicians initiate these conversations and refer patients to appropriate supports. Tools are needed to empower OCPs to confidently discuss PA at any point in the cancer care journey and to identify and capitalize on teachable moments to enhance patients’ quality of life. Given the complexity of care, both individual and system-level factors influence clinician behaviour.

There are specific environmental and systemic barriers to engaging OCPs with supportive services, including a lack of an EMR system to prompt them. The limited success of previous NS Health programs created a feeling of “giving up” on any new or additional programming among health decision makers and OCPs in this study. This idea of “implementation fatigue”, characterized by staff discouragement and resistance after repeated introduction of new initiatives that fail to demonstrate promised benefits, has been reported in cancer care settings [45]. Implementation fatigue can be mitigated by using a bottom-up approach and including OCPs in the implementation and research process. Many OCPs discussed the ease of care if there were an EMR system or tools within their patient records to facilitate discussions about PA. However, PA is seldom seen as a priority within healthcare settings due to the lack of systems supporting it, despite the clear evidence of its benefits. OCPs felt that having easy referral systems would increase the likelihood of PA uptake. This is consistent with previous research that noted patients are receptive to automatic referrals for palliative services in advanced lung cancer [46] and very high acceptance rates for supportive services in the community setting [47]. The use of community-based referral systems within healthcare may be limited due to patient privacy concerns, but they can help bridge gaps between the community and healthcare. It is important to find a way to have meaningful engagement by community partners; however, it is key to balance the barriers of privacy and finding sustainable funding [48]. There are many facilitators to increase the use of a community resources referral system, including embedding the system within workflow, maintaining and updating community resources directories and having strong clinic–community practices [49]. Referral systems are not without their barriers. Cardiac rehabilitation has utilized a referral system within an integrated system; however, physician attitudes, a lack of knowledge about cardiac rehabilitation, and cumbersome referral procedures reduce referral rates. In contrast, strong physician endorsement and an automatic referral system significantly improve patient participation [50]. Exercise oncology practice can draw on valuable lessons from cardiac rehabilitation by starting with a streamlined, automated referral system while conducting widespread and targeted education to ensure OCP endorsement when discussing PA with patients. Helping OCPs overcome systemic barriers and providing them with the physical opportunity to discuss PA may improve equitable access to supportive services.

This research has many strengths, including the use of the COM-B model and TDF, which offer a robust and systematic approach to understanding behavioural drivers. Furthermore, the use of multiple participant groups enables a more comprehensive understanding of PA conversations in clinical care. This study contributes valuable transferability to other settings and contexts. Given specific factors, it could be used outside of the non-curative context, including other chronic conditions or settings [51]. This study is not without limitations, including the use of only one coder, which could lead to subjectivity and bias. However, consultation was conducted during analysis with a senior researcher and a patient partner. Additionally, starting the analysis with deductive methods using the TDF may limit the emergence of novel or underexplored themes.

## 5. Conclusions

The evidence is clear that PA has a positive effect on QOL for those living with non-curative cancer; however, there is a gap in exercise oncology regarding program uptake. This study showed that the use of PA discussion in clinical care can be a great starting point for individuals to find ways to increase and/or maintain their QOL, and that provider support is key for patients to be physically active. Clinicians welcome these conversations in clinical care; however, various barriers limit widespread uptake. Health systems need to provide top-down support and develop creative strategies to integrate PA into clinical care. There is an ongoing need to support individuals living well, which includes not only addressing the immediate medical diagnosis, but also promoting overall health and empowering them to have meaningful engagement in their day-to-day life; however, to do this, we need to empower clinicians to have these conversations about PA effectively, efficiently, and often. Future studies by this team will co-design a tool for providers to use at the point of care to help discuss PA as a supportive and medical service for patients to have in their toolkit.

## Figures and Tables

**Table 1:** TDF specific theme domains with representative quotes.

TDF Domain	Definition According to Michie et al., 2011 [15]	TDF Specific Theme	Representative Quote
Knowledge	Knowledge of the behaviour	Need for integrated PA education in clinical practice	“You know, I think the education piece is really important. We are very siloed and get really focused on how’s the clinic running, when are the patients coming, what is, you know, the evidence behind this treatment plan, and all that stuff. You get really sucked into that. So I think, you know, that communication piece, partly education.” Health Decision Maker 21
Cognitive and Interpersonal Skills	Knowledge and communication skills are required to provide change in behaviour	Influence of patient attitudes on PA discussions	“And then you just kind of… So I must admit, if people don’t look excited, I don’t twist arms. Should we do a bit more of that? There’s enough other things we’re probably twisting arms on… ” OCP 04
Memory, attention and decision processes	The ability to retain knowledge	Challenges with memory recall among OCPs regarding PA	“Yeah. It can be a little thing—“Have discussed, declined,” or… And the date. And so, if six months later, you can say, “I can see six months ago I talked to you about this.” And it could just as well be the nurse or the doctor. Again, the problem is, is anybody going to remember that?” OCP 16
Behavioural Regulation	Anything aimed at managing objectively observed measures of actions	Use of EMR systems to prompt behavioural cues	“You know, you don’t want it on their first visit because they just have so much information that how do you… What’s your trigger to remember on their fifth visit? So you’d need… Could a new EMR, this new ARIA, could it have a trigger—Okay, it’s now six months post, have you thought about referring to X, Y and Z? That would probably work. But I haven’t seen anything like that so far.” OCP 15
Professional/social role and identity	A coherent set of behaviours and displayed personal qualities in an individual in a social and work setting	Value of relational and meaningful roles between patients and providers	“Really cool to be a part of [care for people living with non-curative cancer]. And it’s a very, very, very cool… I mean privileged patient interaction role. Like it’s very special. I appreciate that—getting to know people, getting to spend time with them. We do have time relative to some other specialties. And that’s really important. So you can actually talk to people, and not just sort of address this one, you know, medical issue.” OCP 05
Belief about Capabilities	Acceptance of the truth, reality of validity about an ability, talent or facility that a person can put into constructive use	Empowerment through understanding of self-efficacy	“You mean if I exercise every day, and cut back on what I eat, and do all this exercise stuff, I’m going to get bigger muscles, lose weight? Geez, I have control over that. And it’s not iterative, it’s progressive.” Patient 14
Optimism	The individual will attain the confidence that things will happen for the best or that their desired goals will be attained	Perception of PA as a source of hope in illness management	“So in 2016 the cancer came back in my bones. And, well, I’ve been fighting that with exercise and everything else. And it seems to be working pretty good.” Patient 22
Belief about Consequences	Acceptance of the truth, reality of validity about outcomes of a behavioural change	Anticipation and uncertainty surrounding medical expectations	“I’ve felt good, reasonable, kind of same day every day. Not like the way I was when I had my symptoms last December, but post-procedure I’ve felt the same. And I’m waiting for something to happen. Because that’s what I’ve been told—[Participant], something’s going to happen. But it’s been nine and a half months almost, and it hasn’t happened yet.” Patient 20
Intentions	A conscious decision to perform or not perform a behaviour or resolve to act in a certain way	Recognition that formal exercise programs may not suit everyone, but alternative forms of PA are valuable	“Dear God, no. [laughs] Physical activity, no. Yeah, I mean I go on walks with [patient]. I probably would not go for walks if it were not for [patient]. Because as I said, I’m not… Yeah, I enjoy being outside and doing things. But yeah, for me to join [exercise program] or something like that, or go to aerobics, that probably would never have happen, no.” Caregiver 17
Goals	Identification of goals and responsibilities attached to their role	Health scares as triggers for sustained behaviour change	“Well, that’s sickened me too when I got in that pool and I almost drowned. I said, I’ve got to do something about this.” Patient 10
Environmental Context and Resources	Any factors in the environment that encourage the behaviour	Desire for streamlined and coordinated healthcare systems	“Like I said, if you just like click a button. So it’d be great if like ARIA could have an [exercise] referral built right into it. Those kinds of things as like longer term goals.” Health Decision Maker 12

ARIA: Trade name for an Oncology Information Software for electronic medical records that NS Health was trying to implement at the time of this study, EMR: Electronic Medical Records, OCP: Oncology Care Providers.

**Table 2:** Overarching themes that align with the COM-B model.

Psychological Capability	Reflective Motivation	Physical Opportunity
Effective communication and targeted education are essential to addressing knowledge gaps about PA. These targeted education programs can help influence beliefs, supporting informed decision making, thoughtful engagement, and behaviour change. They can also be effective in two ways: (1) health decision makers influencing OCPs, and (2) OCPs influencing patients.	There is an understanding that emotional barriers can impact engagement with PA. OCPs and health decision makers are motivated to address these challenges by prioritizing process improvements and support strategies that foster motivation and make PA more accessible and meaningful for patients.	There are identified environmental and systemic barriers to engaging patients with PA supports, and an emphasized need for streamlined or integrated referral processes to enhance accessibility and allow patients to start PA at any point of their cancer care journey.

## Data Availability

The data presented in this study are available on request from the corresponding author. The data are not publicly available due to ethical restrictions and small sample size making individuals potentially identifiable.

## Abbreviations

The following abbreviations are used in this manuscript:

COM-B	Capability, Opportunity, Motivation–Behaviour
OCP	Oncology Care Provider
PA	Physical Activity
QOL	Quality of Life
TDF	Theoretical Domains Framework
